# Lysis of *Microcystis aeruginosa* with Extracts from Chinese Medicinal Herbs

**DOI:** 10.3390/ijms10094157

**Published:** 2009-09-23

**Authors:** Jing-Dong Yang, Liang-Bin Hu, Wei Zhou, Yu-Fen Yin, Jian Chen, Zhi-Qi Shi

**Affiliations:** 1 Key Lab of Food Quality and Safety of Jiangsu Province, Jiangsu Academy of Agricultural Sciences, Nanjing 210014, China; E-Mails:smartforest@yahoo.cn (J.D.Y.);zhouwei@jaas.ac.cn (W.Z.);yingyu-2006@163.com (Y.F.Y.);chenjian@jaas.ac.cn (J.C.); 2 School of Food, Henan Institute of Science and Technology, Xinxiang 453003, China; E-Mail:hlb197988@163.com (L.B.H.)

**Keywords:** Chinese medicinal herbs, Microcystis aeruginosa, cyanobacteria, lysis

## Abstract

Boiling water extracts of 66 selected Chinese medicinal herbs were screened for their anticyanobaterial activity against *Microcystis aeruginosa* by the soft-agar overlayer (SAO) method. Results indicated that extracts from 16 materials could inhibit the growth of this bacterial species. Among these anticyanobacterial samples, eight extracts showed low minimum inhibitory concentrations (MIC), including four extracts with MICs between 1 and 6 mg/mL, and four extracts with MICs < 1 mg/mL which could be considered useful to prevent the outbreak of cyanobacteria before the appearance of cyanobacterial blooms. Further study showed that three extracts with MIC values < 1 mg/mL induced intensive chlorophyll-a lysis within 7 days at the MIC. The results suggested that highly efficient anticyanobacterial compounds must be involved in the inhibitory activities. The final results indicated these three extracts (from *Malaphis chinensis, Cynips gallae-tinctoriae* and *Fructus mume*) had the potential to be developed as algicides due to their remarkably anticyanobacterial activities.

## 1. Introduction

Cyanobacteria (blue-green algae) are photoautotrophic Gram-negative bacteria which commonly evoke occurrence of blooms and scums in lakes, reservoirs, slow-flowing rivers [1]. Due to their musty odor and production of potent toxins, cyanobacteria populations are a great concern in reservoir supplies and recreational water systems [2]. *Microcystis aeruginosa*, found globally in fresh waters, is a cyanobacterium which can produce toxins threatening public health [3], so *M. aeruginosa* has been the subject of increasing research over the last decades. To minimize the threat, methods of prevention and control for bloom problems have been adopted, such as chemical treatments with algaecides [4] and biological control [5]. Algicidal compounds are widely used in industrial waters, for the sanitation of swimming pools and the like. Copper sulfate, chelated copper compounds, and diuron (3-[3,4-dichlorophenyl]-1,1-dimethylurea) are the only compounds currently approved by the U.S. Environmental Protection Agency for use as algicides in catfish production ponds. Unfortunately, these compounds have the following undesirable characteristics: (i) broad-spectrum toxicity towards phytoplankton that can result in the death of the entire phytoplankton community and subsequent water quality deterioration that may stress or kill catfish; (ii) lengthy persistence in the environment that creates concerns about environmental safety; and (iii) the public’s negative perception of the use of synthetic herbicides in food fish production ponds [6]. Ideally, in the concentration employed, they should be harmless for man and animals.

Thousands of plants worldwide are used in traditional medicine as treatments for bacterial infections and especially traditional Chinese medicinal herbs which contain abundant potential antimicrobial agents have been used for the treatment of a wide variety of diseases for thousands of years [7]. In recent years, screening of plant resources for antimicrobial compounds has being intensively carried on worldwide, but there has been no screening of Chinese herbs extracts against *M. aeruginosa.*

In this study, we aimed to investigate potential useful plant compounds or extracts from Chinese herbs which should ideally display lysis activity against cyanobacteria. We expect that this study will contribute to the control of pollution by *M. aeruginosa* and the following identification of active compounds for emergencies caused by this species.

## 2. Results and Discussion

### 2.1. Results

#### 2.1.1. Anticyanobacterial Screening

By the SAO method, diverse levels of anticyanobacterial activities were observed in the plates containing different tested extracts. The final screening results are listed in Table 1. Generally, it was found that extracts from 16 materials showed inhibitory activity towards *M. aeruginosa*. Among the 16 extracts, *Herba Patriniae*, *Forsythia suspensa* (Thunb.) Vahl, *Rubia cordifolia*, *Polygala tenuifolia*, *Acorus tatarinowii*, *Sophora flavescens*, *Rhizoma Chuanxiong*, *Rhizoma Corydalis* and *Ranunculus ternatus* extracts showed low inhibition levels to *M. aeruginosa* with DIZ (diameter of inhibition zone) values ≥ 10 mm; *Fraxinus rhynchophylla, Crataegus pinnatifida,* and *Euphorbia humifusa* extracts showed moderate levels of activity, with DIZ values ≥ 20 mm; *Cornus officinalis* Sieb. et Zucc*, Malaphis chinensis, Cynips gallae-tinctoriae* and *Fructus mume* extracts showed high activity levels, withDIZ values ≥ 30 mm.

#### 2.1.2. Determination of *M. aeruginosa*-Inhibiting Abilities of 16 Selected Chinese Herbs Extracts

The inhibitory abilities of selected Chinese herbs extracts were confirmed by determining the MIC towards *M. aeruginosa*. All the results wre listed in Table 2. Extracts of *C. gallae-tinctoriae, M. chinensis, F. mume, Herba Patriniae* exhibited the lowest MIC values (<1 mg/mL); The MIC values of *C. pinnatifida, C. officinalis Sieb. Et, F. rhynchophylla, E. humifusa* are all at 3.125 mg/mL. MIC values of the remaining eight extracts were all higher than 6 mg/mL, which meant their *M. aeruginosa*-inhibiting abilities were very low and so they were excluded in the sebsequent study.

#### 2.1.3. Dynamic Analysis of Chlorophyll-a in *M. aeruginosa* with Remained 8 Chinese Herbs Extracts

**Figure 1.**
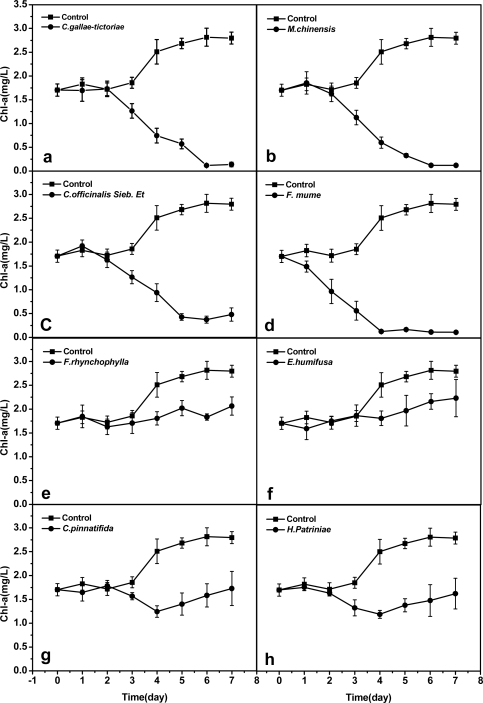
Effects of different Chinese herbs extracts on the contents of chlorophyll a at MIC value. Control: *M.aeruginosa* (▪); Treat: Chinese herbs extracts (•) [a: *C.gallae-tictoriae* (0.39 mg/mL); b: *M.chinensis* (0.39 mg/mL); c: *C.officinalis Sieb. Et* (3.125 mg/mL); d: *F. mume* (0.78 mg/mL); e: *F.rhynchophylla* (3.125 mg/mL); f: *E.humifusa* (3.125 mg/mL); g: *C.pinnatifida* (3.125 mg/mL); h: *H. Patriniae* (0.78 mg/mL)]. Each point represents mean ±SE of three replications.

Changes of the chlorophyll a (Chl-a) contents of the *M. aeruginosa* in a sterile BG11 culture solution for seven days were recorded as a time course curve (Figure 1). Effects of the eight finally selected Chinese herbs extracts on the *M. aeruginosa* growth could be observed from corresponding curve. Generally speaking, each extract finally caused the Chl-a lysis of *M. aeruginosa*, but their lysing actions differed with regards to the inception time and efficacy. From Figure 1, it is seen that except for treatment with *P. mume* extract, the Chl-a of all treated *M. aeruginosa* started lysing two days after the inoculation of tested extracts. *C. gallae-tictoriae*, *M. chinensis*, *C.officinalis Sieb. Et* and *F. mume* extracts almost induced Chl-a complete lysis in seven days (Figures 1 a–d). However, *F. rhynchophylla*, *E. humifusa*, *C. pinnatifida* and *H. Patriniae* extracts only induced partial lysis of Chl-a in seven days (Figures 1 e–h).

### 2.2. Discussion

China has a rich flora that is widely distributed throughout the country. Chinese medicinal herbs have been the basis of treatment and cure for various diseases and physiological conditions in traditional methods. In fact, many naturally occurring compounds found in plants, herbs, and spices have been shown to possess antimicrobial functions and serve as sources of antimicrobial agents against foodborne pathogens [8]. In this study, it was indicated that many extracts from selected Chinese medicinal herbs possessed ideal inhibition activity against cyanobacteria.

The SAO method was employed for screening the anticyanobacterial Chinese herbs. Anticyanobacterial activity was evaluated by measuring the DIZ of the tested cyanobacterium. In such tests, extracts containing active insolvable compounds would escape detection, but considering the potential applications, it was preferred to focus on the extracts containing active soluble compounds. Solubility and diffusion of active components in agar media could play a major role in the formation of inhibition zone, which suggested that DIZ might not be absolutely equivalent to the anticyanobacterial capability, hence the MIC values of all positive extracts screened by SAO were determined to evaluate their anticyanobacterial capability. Extracts of *C. gallae-tinctoriae*, *M. chinensis*, *F. mume*, and *H. Patriniae* exhibited low MIC values (<1 mg/mL), which indicated their potential to be timely applied to prevent the outbreak of cyanobacteria before the occurrence of cyanobacterial bloom.

The *M. aeruginosa*-lytic activity of the selected extracts was evaluated for their potential as algicides. It was found that extracts of *C. gallae-tinctoriae, M. chinensis, F. mume* and *C. officinalis Sieb. Et* showed obvious lytic activity towards *M. aeruginosa*, while extract of *H. Patriniae* showed too weak lytic activity to be a useful algicide. Considering the far higher MIC and average *M. aeruginosa*-lytic activity, the extract of *C.officinalis Sieb Et* was not recommend for development as an algicide. It should be noted that the initial time for lytic actions of these extracts were different at their MICs, and *F.mume* extract exhibited fast acting lysis activity against Chl-a of *M. aeruginosa* which was different from other extracts. These results suggested that *P. mume* extract probably contained some special active compounds involved in the lysis of *M. aeruginosa*, and such extract could be considered as acute algaecide applied in some emergencies caused by *M. aeruginosa*.

Many volatile organic compounds have been found to have lytic activity against cyanobacteria. It was confirmed that volatile terpenoid compounds produced by plants had lytic activity [9]. According to Cowan [10], aqueous plant extracts mainly contain anthocyanins, starches, tannins, saponins, terpenoids, polypeptides, lectins, etc, so Chinese herbs extracts could be expected to have universal anticyanobacteria activity, but this was not really the case. Many extracts did not display any anticyanobacteria activity. Therefore, it was implied that the content of the active ingredients was different in different Chinese medicinal herbs. For instance, the aqueous extracts of *F. mume* mainly contain organic acids, terpenoids, sterols, flavonoids, carbohydrates and amino acids. Especially, the concentration of organic acids was very high (up to 40.5%) [11]. Malic acid and citric acid were the major organic acid constituents, while ethanedioic acid, glycolic acid, lactic acid, succinic acid, formic acid, acetic acid, propionic acid, etc were also present. Lots of these organic acids have been reported to have inhibition activity towards some microbe species [12–14], so it was very likely that these organic acids induce the lysis of cyanobacteria. Of course, terpenoids (ursolic acid) and flavonoids may show synergistic effect on the growth of cyanobacteria. In addition, some amino acids (*e.g.*, lysine, histidine, alanine) may also accelerate the lysis of cyanobacteria [15]. *M. chinensis* is a traditional Chinese herb distributed widely in southern China. It is the gallae that is produced by some parasitic aphids (family Pemphigidae) on Rhus leaves of the family Anacardiaceae (mainly *Rhus chinensis* Mill, *Rhus potaninii* Maxim, and *Rhus punjabensis* var. sinica (Diels) Rehd. et Wils) [16]. The surface feature of *C. gallae-tinctoriae* is very similar to *M. chinensis*. Both of them are gallae, and the active compounds from their aqueous extracts should be also similar, containing a large amount of gallotannin, a typical hydrolysable tannin, with the content of up to 70% of its weight [17]. The content of gallic acid in the extract is about 2%~4%. It was reported that gallotannin and gallic acid could inhibit the growth of intestinal bacteria [18], so it was presumed that gallotannin and gallic acid were at least partially responsible for the observed lysis of cyanobacteria by the extracts from *M. chinensis* or *C. gallae-tinctoriae*. Even the active extracts showed diverse levels in efficiency and inception time. The results indicated that multiple and diverse active compounds with anticyanobacteria activity existed in the extracts. It would be of interest to purify and identify the responsible bioactive components from these extracts.

## 3. Experimental Section

### 3.1. Cyanobacteria Culture and Chinese Medicinal Herbs

*M.aeruginosa* (FACHB 905) was obtained from the Freshwater Algae Culture Collection of the Institute of Hydrobiology (FACHB), located in China. The strain was grown in BG-11 medium [19] at 25 °C with illumination at 3,000 lx under a 16 L/8 D cycle. Sixty-six Chinese medicinal herbs were obtained from traditional medicine stores in Nanjing. The different parts of the plant used were the leaves, the branches, the fruits, the rhizomes, the peels, the gallaes shown in Tables 1 and 2.

### 3.2. Preparation of the Extracts

Stock solutions were prepared with the traditional process currently used in Chinese clinics and scientific studies by boiling 100 g of raw material with 1,000 mL of distilled water for 1 h. The material was centrifuged and filtered through filter paper. The residue remaining on the filter paper was reboiled with 1,000 mL distilled water, centrifuged, and refiltered. The resulting two batches of the solution were mixed and then boiled again until 100 mL remained. This solution was regarded as a concentration of 100% (100 mL of HC solution made from 100 g of raw material). After being autoclaved, the stock solution was stored at 4 °C until used.

### 3.3. Measurement of Anticyanobacterial Activity

The anticynobacterial activity was determined using the soft-agar overlayer (SAO) method [20]. Approximately 5 mL of cyanobacterial cells (2 × 10^6^ cells/mL) were mixed with warmed 5 mL of 0.8% (w/v) soft agar and over-layered on a 10 mL of 1.2% (w/v) agar layer solidified in a plate. After the cyanobacterium containing layer was solidified. Sterilized Oxford cups (8 mm in external diameter) were put up on the cyanobacterium-containing layer regularly and 200 μL extract solutions were respectively deposited on the disc, and the concentration of the test herb extracts were diluted to 200 mg/mL. 20 μg/mL CuSO_4_ and distilled water were used as positive and negative controls, respectively. The plates were incubated at 25 °C for 2 days. Anticyanobacterial activity was evaluated by measuring the diameter of inhibition zone (DIZ) of the tested cynobacterium. DIZ was expressed in millimeters. All tests were performed in triplicate.

### 3.4. Determination of Minimum Inhibitory Concentration (MIC)

Several anticynobacterial extracts were screened from the material extracts by disc diffusion method. A broth microdilution method was used to determine the MIC [21,22]. An aliquot of 0.1 mL of a serially diluted Chinese herb extracts in BG11 medium was added to a 96-well microplate and 0.1 mL of cyanobacteria cultured broth (2 × 10^6^ cells/mL) was added to each well. Then the resulting solution was incubated at 25 °C under 3,000 lx continuous illumination for a week. The MIC is defined as the lowest concentration of the extracts at which the cynobacteria does not demonstrate visible growth. Each test was performed in triplicate.

### 3.5. Extraction and Measurement of Cyanobacterial Chlorophyll a

A solution of 90% methanol was used for extraction of chlorophyll a (chl-a) from cyanobacteria. Measurement of chl-a was according to the method of Parsons and Strickland [23]. Cyanobacteria collected by centrifugation (12,000 r.p.m., 15 min, 4 °C) were subjected to extraction with 90% methanol in a water bath (60 °C) for 10 min. After extraction, the solid suspension was removed by centrifugation (12,000 r.p.m., 15 min, 4 °C). Then, the absorbance of extracts at 665, 645 and 635 nm was measured using a spectrophotometer (Perkin Lambda 25). The concentration of chl-a was calculated using the following equation [15]: Chl-a (μg/mL) = 11.6A_665_-1.31A_645_-0.14A_635_.

## 4. Conclusions

In this study, it was indicated that many Chinese herbs possess anticyanobanteria activity. Our results showed that *C. gallae-tinctoriae*, *M. chinensis*, and *F. mume* have potential as algicides due to their remarkable anticyanobacterial effects.

## Figures and Tables

**Table 1.** Anticynobacterial activity of various extracts with boiling water by the disc diffusion method.

**Botanical name**	**English name**	**Part tested**	***M.aeruginosa***
Control		N	−
		P	++
*Cornus officinalis* Sieb. et Zucc	Medical Dogwood	Fruit	+++
*Malaphis chinensis*	Gallnut	Gallae	+++
*Fructus mume*	Smoked Plum	Fruit	+++
*Cynips gallae-tinctoriae* Olivier	Nutgall	Gallae	+++
*Euphorbia humifusa* Willd	Humifuse Spurge	Whole plant	++
*Fraxinus rhynchophylla* Hance	Ash Bark	Peel	++
*Crataegus pinnatifida*	Hawthorn	Fruit	++
*Rubia cordifolia* Linn.	India Madder Root	Whole plant	+
*Polygala tenuifolia* Willd	Thinleaf Milkwort Root	Whole plant	+
*Acorus tatarinowii* Schott	Rhizoma Acori Tatarinowii	Whole plant	+
*Sophora flavescens* Alt	Lightyellow Sophora Root	Rhizome	+
*Rhizoma Chuanxiong*	Ligusticum Chuanxiong Hort	Rhizome	+
*Rhizoma Corydalis*	Yanhusuo	Whole plant	+
*Forsythia suspensa* (Thunb.) Vahl	Weeping Forsythia	Whole plant	+
*Herba Patriniae*	White flower Patrinia Herb	Whole plant	+
*Ranunculus ternatus* Thunb	Radix Ranunculi Ternati	Whole plant	+
*Eichhornia crassipes* (Mart.) Solms	Weter Hyacinth	Branch	−
*Suaeda glauca* Bge	Common Seepweed Herb	Whole plant	−
*Houttuynia cordata* Thuhb	Herba Houttuyniae	Whole plant	−
*Isatis tinctoria* L	Isatis Root	Rhizome	−
*Herba Taraxaci*	Dandelion	Whole plant	−
*Pulsatilla chinensis* (Bunge) Regel	Anemone	Whole plant	−
*CoptischinensisFranch*	Coptis Chinensis	Whole plant	−
*Folium Isatidis*	Folium Isatidis	Leaf	−
*Prunella vulgaris*	Spica Prunellae	Whole plant	−
*Punica granatum*	Pomegranate	Peel	−
*Terminalia chebula* Retz	Medicine Terminalia Fruit	Fruit	−
*Gardenia: jasminoides* Ellis	Cape Jasmine	Fruit	−
*Viola philippica ssp.munda* W. Beck	Purple flower Violet	Leaf	−
*Anemarrhena asphodeloides* Bunge	Common Rhizoma Anemarrhenae	Fruit	−
*Rhizoma Cyperi*	Nutgrass Galingale Rhizom	Fruit	−
*Lithospermum erythrorhizon* Sieb.et Zucc	Gromwell Root	Whole plant	−
*Herba Artemisiae* Annuae	Sweet Wormwood Herb	Whole plant	−
*Zanthoxylum bungeanum*	Pricklyash Peel	Fruit	−
*Aucklandia lappa* Decne.	Radix Aucklandiae	Rhizome	−
*Glycyrrhiza uralensis* Fisch	Licorice Roots Northwest Origin	Whole plant	−
*Rhizoma Smilacis* Glabrae	Glabrous Greenbrier Rhizome	Rhizome	−
*Sanguisorba officinalis* Linn	Garden Burnet	Rhizome	−
*Pericarpium Citri* Reticulatae	Dried Tangerine peel	peel	−
*Herba Senecionis Scandentis*	Climbing Groundsel Herb	Whole plant	−
*Schisandra chinensis* (Turcz.) Baill	Chinese Magnolivine Fruit	Fruit	−
*Clematis chinensis* Osbeck	Radix Clematidis	Whole plant	−
*Drynaria fortunei* (Kunze) J. SM	--	Rhizome	−
*Benincasa hispide* Thunb	White gourd	Peel	−
*Astragalus membranaceus* (Fisch.) Bunge	Astragali	Rhizome	−
*Bupleurum chinense* DC	Bupleuri	Rhizome	−
*Geranium wilfordii* Maxim	Herba erodii	Whole plant	−
*Subgen. Tsutsusi* (G. Don) Pojarkova	Loquat	Leaf	−
*Cortex Cinnamomi* Cassiae	Cinnamon	Rhizome	−
*Andrographis paniculata*(Burm.f.) Nees	Common Andrographis Herb	Whole plant	−
*Lonicera japonica* Thunb	Flos Lonicerae	Flower	−
*Tradescantia albiflora*	--	Whole plant	−
*Portulaca oleracea* Linn	Purslane	Whole plant	−
*Plantago asiatica* L	plantain	Whole plant	−
*Trachelospermum jasminoides* (Lindl.)Lem	Caulis Trachelospermi	Whole plant	−
*Semen Cassiae*	Cassia Seed	Seed	−
*Pogostemon cablin* (Blanco) Benth	Agastache rugosa	Whole plant	−
*Folium llicis* Latifoliae	Broadleaf Holly leaf	Leaf	−
*Cyrtomium fortunei* J. Sm	Cyrtomii Rhizoma	Rhizome	−
*Herba Menthae* Heplocalycis	Wild Mint Herb	Leaf	−
*Syzygium aromaticum* (L.) Merr. Et Perry	Flos Caryophyllata	Leaf	−
*Platycladus orientalis* (Linn.)Franco	Arborvitae	Leaf	−
*Magnolia liliiflora* Desr	Flos Magnoliae	Fruit	−
*Areca catechu* Linn	Betel nut	Peel	−
*Dryobalanops aromatica* Gaertn. f.	Borneol	Resin	−
*Sterculia lychnophera* Hance	Boat-fruited Sterculia Seed	Fruit	−
*Gynostemma pentaphyllum* Thunb. Makino	Fiveleaf Gynostemma Herb	Leaf	−
*Reynoutria japonica* Houtt	Rhizoma Polygoni Cuspidati	Leaf	−

*Abbreviations*: N, negative control (distilled water); P, positive control (CuSO4 20 μg/mL); Grading of results: +++, complete inhibition (DIZ: 30~40 mm); ++, moderate inhibition (DIZ: 20~30 mm); +, partial inhibition (DIZ: 10~20 mm); –, no inhibition (DIZ: 8 mm); The outside diameter of oxford cup on the soft-agar overlayer is 8 mm and the diameter of inhibition zone (DIZ) of negative control is also 8 mm. If the DIZ value is 8 mm, that means the extract has no inhibitory activity against *M. aeruginosa*.

**Table 2.** MIC values of several chinese herbs against the growth of *Microcystis aeruginosa*.

**Botanical name**	**English name**	**Part tested**	**MIC (mg/mL)**
*Cynips gallae-tinctoriae* Olivier	*Nutgall*	Gallae	0.39
*Malaphis chinensis*	*gallnu*	Gallae	0.39
*Herba Patriniae*	*White flower Patrinia Herb*	Whole plant	0.78
*Fructus mume*	*Smoked Plum*	Fruit	0.78
*Crataegus pinnatifida*	*Nippon Hawthorn Fruit*	Fruit	3.125
*Cornus officinalis Sieb. et* Zucc	*Medical Dogwood*	Fruit	3.125
*Fraxinus rhynchophylla* Hance	*Ash Bark*	Peel	3.125
*Euphorbia humifusa* Willd	*Humifuse Spurge*	Whole plant	3.125
*Forsythia suspensa (Thunb.)* Vahl	*Weeping Forsythia*	Whole plant	6.25
*Polygala tenuifolia* Willd	*Thinleaf Milkwort Root*	Whole plant	6.25
*Rubia cordifolia* Linn.	*India Madder Root*	Whole plant	6.25
*Ranunculus ternatus* Thunb	*Radix Ranunculi Ternati*	Whole plant	12.5
*Acorus tatarinowii* Schott	*Rhizoma Acori Tatarinowii*	Whole plant	12.5
*Rhizoma Chuanxiong*	*Ligusticum Chuanxiong Hort*	Rhizome	12.5
*Sophora flavescens* Alt	*Lightyellow Sophora Root*	Rhizome	25
*Rhizoma Corydalis*	Yanhusuo	Whole plant	25
